# Cut-off points of neck and waist circumference as predictors of obstructive sleep apnea in the Colombian population: a comparison with polysomnography

**DOI:** 10.1590/1516-3180.2022.0415.R2.310523

**Published:** 2023-12-04

**Authors:** Sandra Brigitte Amado Garzon, Oscar Mauricio Muñoz-Velandia, Alvaro J Ruiz, Patricia Hidalgo Martínez, Liliana Otero

**Affiliations:** I MD, MSc. Assistant Professor, School of Medicine, Pontificia Universidad Javeriana, Bogotá, Colombia; and Internist, Department of Internal Medicine, Hospital Universitario San Ignacio, Bogotá, Colombia.; II MD, PhD. Associate Professor, School of Medicine, Pontificia Universidad Javeriana, Bogotá, Colombia; and Internist, Department of Internal Medicine, Hospital Universitario San Ignacio, Bogotá, Colombia.; III MD, MSc. Titular Professor, Department of Clinical Epidemiology and Biostatistics, School of Medicine, Pontificia Universidad Javeriana, Bogotá, Colombia.; IV MD, MSc. Titular Professor, Department of Internal Medicine, School of Medicine, Pontificia Universidad Javeriana, Bogotá, Colombia; Pulmonologist, Sleep Clinic, Department of Internal Medicine, Pulmonology Unit, Hospital Universitario San Ignacio, Bogotá, Colombia.; V DDS, MSc, PhD. Titular Professor, Department of Craniofacial System, School of Dentistry, Pontificia Universidad Javeriana, Bogotá, Colombia.

**Keywords:** Waist circumference, Sleep apnea, obstructive, Area under curve, Body mass index, Obstructive apnea, Cut-off point, Neck circumference

## Abstract

**BACKGROUND:**

Neck circumference (NC) is a useful anthropometric measure for predicting obstructive sleep apnea (OSA). Ethnicity and sex also influence obesity phenotypes. NC cut-offs for defining OSA have not been established for the Latin American population.

**OBJECTIVES:**

To evaluate NC, waist circumference (WC), and body mass index (BMI) as predictors of OSA in the Colombian population and to determine optimal cut-off points.

**DESIGN AND SETTING:**

Diagnostic tests were conducted at the Javeriana University, Bogota.

**METHODS:**

Adults from three cities in Colombia were included. NC, WC, and BMI were measured, and a polysomnogram provided the reference standard. The discrimination capacity and best cut-off points for diagnosing OSA were calculated.

**RESULTS:**

964 patients were included (57.7% men; median age, 58 years) and 43.4% had OSA. The discrimination capacity of NC was similar for men and women (area under curve, AUC 0.63 versus 0.66, P = 0.39) but better for women under 60 years old (AUC 0.69 versus 0.57, P < 0.05). WC had better discrimination capacity for women (AUC 0.69 versus 0.57, P < 0.001). There were no significant differences in BMI. Optimal NC cut-off points were 36.5 cm for women (sensitivity [S]: 71.7%, specificity [E]: 55.3%) and 41 cm for men (S: 56%, E: 62%); and for WC, 97 cm for women (S: 65%, E: 69%) and 99 cm for men (S: 53%, E: 58%).

**CONCLUSIONS:**

NC and WC have moderate discrimination capacities for diagnosing OSA. The cut-off values suggest differences between Latin- and North American as well as Asian populations.

## INTRODUCTION

Obstructive sleep apnea (OSA) is highly prevalent among adults. Approximately 425 million (399–450) adults, aged 30–69 years have moderate to severe obstructive sleep apnea globally, with more affected individuals in China, followed by the United States, Brazil, and India.^1^ In patients with cardiovascular disease, the prevalence rises to 40%–60%.^2^ Additionally, the risk of fatal and non-fatal cardiovascular events is significantly higher in patients with severe untreated OSA (adjusted odds ratio, OR 2.8 for fatal and 3.1 for non-fatal).^3^

Neck circumference (NC) is one of the screening strategies useful for predicting OSA.^4^ For this reason, NC is included in widely used questionnaires such as the STOP Bang,^5^ which proposes an NC cut-off point of 40 cm. NC is associated with fat deposition in the anterolateral region of the upper airway.^6^ Its measurement complements body mass index (BMI) information and correlates with OSA severity, regardless of visceral fat, especially in non-obese patients.^7^

In the North American population, NC larger than 17 inches (43 cm) in men and 16 inches (41 cm) in women has been strongly associated with the risk of OSA.^8^ These values are notably higher than the values proposed for Asian populations (38.75 cm in men and 34.5 cm in women).^9^ These differences suggest the existence of various obesity phenotypes, partly explained by ethnic factors that also account for geographic differences in OSA prevalence.^10,11^ Reported differences between North American and Asian populations highlight the importance of establishing specific cut-off points for each population. In Latin America, there is limited information on anthropometric measurements, prompting the need to determine optimal NC cut-off points that may help to predict OSA.^12^

Similarly, the optimal waist circumference (WC) cut-off point for predicting OSA in Latin America is unknown. Previous studies have proposed cut-off points of 94 cm for men and 90–92 cm for women as markers of visceral adiposity,^13,14^ but the value of these measurements in predicting OSA is currently unknown.

Although previous studies in Latin America^15^ have described a possible association between sleep disorders and anthropometric measurements, none have compared these measurements with the existing gold standard of polysomnography (PSG).

## OBJECTIVE

The goal of this study was to evaluate NC, WC, and BMI as screening tests to identify patients with OSA, and to compare anthropometric measurements with PSG results. This study also aimed to determine the optimal cut-off points for these measurements in a representative sample from three Colombian regions.

## METHODS

This multicenter study aimed to assess screening tests and determine cut-off points. We included adults older than 18 years from three cities in Colombia (Bogotá D.C., Santa Marta, and Bucaramanga) who attended primary or secondary prevention programs at the Instituto del Corazón, as well as patients from the general population of those cities who accepted an invitation to participate in the study. Patients with mental illnesses that could interfere with data collection were excluded. The Committee of Ethics and Research of the Pontificia Universidad Javeriana in Bogotá, D.C. (Colombia) approved the research protocol (M-CIE 55761, January 26, record 1-2012).

Each participant completed a form that included questions on demographics and comorbidities along with several screening questionnaires, including the Epworth Sleepiness Scale, Berlin Questionnaire, and Pittsburgh Sleep Quality Index. Patients with scores above the established cut-off points were candidates for PSG.

A trained professional used a nonelastic measuring tape and followed standardized techniques to obtain anthropometric measurements, including body weight, height, NC, and WC. WC was measured at the mid-axillary line, midway between the lowest rib and the iliac crest, with the patient in expiration, as recommended by the International Diabetes Federation.^16^ NC was measured at the cricothyroid membrane level; and BMI was established according to World Health Organization (WHO) parameters.^17^

Immediately after the anthropometric measurements, participants underwent conventional type 1 PSG with Alice 5 (Philips Respironics, Murrysville, United States) from 9 p.m. to 6 a.m. The study included readings from nasal and oral flow transducers, a snore microphone, video recordings, electroencephalograms, electrooculograms, electromyograms, electrocardiograms, belts for thoracic and abdominal effort impedance measurements, pulse oximetry, and sensors for position changes during sleep. One certified sleep specialist interpreted all the tests, according to the American Academy of Sleep Medicine (AASM) parameters.^18^ This second evaluator was blinded to the results of the screening questionnaires and the anthropometric measurements. The AASM hypopnea criteria require a 30% reduction in airflow accompanied by a 4% oxygen desaturation or a 50% reduction in airflow accompanied by a 3% oxygen desaturation or arousal. Apnea was defined as the cessation of airflow for at least 10 s. Criteria for diagnosing OSA were an Apnea Hypopnea Index (AHI) > 15 with no symptoms, or ≥ 5 in the presence of symptoms. The severity of the condition was classified according to AHI values as mild (AHI >5 and < 15 per h), moderate (AHI > 15 and < 30 per h), or severe (AHI > 30 per h).^19^

### Statistical analysis

The Shapiro-Wilks test was used to assess variable normality. Averages and standard deviations were used for numerical variables with a normal distribution, and medians with interquartile ranges were used for variables with non-normally distributed data. Groups were compared using an unpaired two-tailed t-test, chi-square test, or a Mann-Whitney U test, according to the type of variable.

Sensitivity (S) and specificity (E) were calculated for the NC, WC, and BMI cut-off points and compared to the PSG results as a reference standard. Receiver operating characteristic (ROC) curves were differentially calculated by sex and age group (younger and older than 60 years), and the areas were compared using a nonparametric approach.^20^

The study determined optimal cut-off points for each measurement using the Liu method.^21^ Additionally, NC operative characteristics were presented with a 40 cm cut-off point, taking into account values used in the STOP-Bang questionnaire.^5^ For WC, a 90 cm cut-off point was established according to previous studies in the Colombian population.^13^ For BMI, the cut-off point was set at 30 according to WHO recommendations for diagnosing obesity.^17^

Data were analyzed using the STATA statistical package 14.0 (Stata Corp, College Station, Texas, United States).

## RESULTS

The study included 964 patients, mostly men (58%) aged between 18 and 91 years of age. Median ages were 59 years for men and 57 years for women. Most of the participants were overweight, with a median BMI of 26; obesity prevalence was 18.7% in men and 23.1% in women. At assessment, 65.9% of patients had cardiovascular disease, defined as heart disease of any cause, coronary artery disease, or cerebrovascular disease.

Regarding comorbidities, high blood pressure (HBP) was most frequent (57%), followed by coronary disease (44%) and diabetes mellitus (16%). Of patients with HBP, 15% had AHI >30. Hypothyroidism and diabetes were more frequent in patients with HBP. **Table 1** describes the clinical and demographic characteristics of the study population according to sex.

**Table 1:** Sociodemographic characteristics of included patients

	Women	Men	P value
	n (%)	n (%)	
	408 (42.3)	556 (57.7)	
**Age in years, median (IQR)**	57 (45–67)	59 (51–69)	0.01
**BMI, Kg/m^2^, n (%)**			
< 18	0 (0)	3 (0.5)	0.08
18–24.9	149 (36.5)	190 (34.2)	
25–29.9	165 (40.4)	259 (46.6)	
> 30	94 (23.1)	104 (18.7)	
**Neck circumference in cm, median (IQR)**	37 (35–48)	41 (39–44)	< 0.001
**Neck circumference in cm, mean (SD)**	37.2 (3.4)	41.1 (3.5)	< 0.001
**Waist circumference in cm, median (IQR)**	98 (89–106)	99 (92–109)	< 0.001
**Waist circumference in cm, average (SD)**	97 (13.16)	99 (10.9)	< 0.001
Snoring*, n (%)			
**Yes**	260 (63.7)	388 (69.8)	0.015
**No**	77 (18.9)	107 (19.2)	
**Don´t know**	71 (17.4)	61 (11.0)	
Snoring loudly^**^ , n (%)	140 (34.1)	239 (43.0)	0.006
***OSA, n (%)	**173 (42.4)**	**245 (44.1)**	
Mild	99 (24.3)	113 (20.3)	0.599
Moderate	47 (11.5)	73 (13.1)	
Severe	27 (6.6)	59 (10.6)	
**Comorbidities n (%)**			
Coronary disease	134 (32.8)	289 (52.0)	< 0.001
HBP	216 (52.9)	332 (59.7)	0.035
Diabetes	55 (13.5)	97 (17.4)	0.101
Depression	76 (18.6)	60 (10.8)	0.001
Anxiety	72 (17.6)	64 (11.5)	0.007
Hypothyroidism	93 (22.8)	56 (10.1)	< 0.001
COPD	19 (4.6)	23 (4.1)	0.705
GERD	49 (12.0)	60 (10.8)	0.561

IQR = interquartile range; BMI = body mass index; SD = standard deviation; OSA = obstructive sleep apnea; HBP = high blood pressure; COPD = chronic obstructive pulmonary disease; GERD = gastro esophageal reflux disease.

* According to the question, Do you snore? included in the Berlin questionnaire;

** According to the question Do you snore loud? Included in the STOP-BANG questionnaire;

** Results by polysomnogram (PSG).

A total of 44.1% of men and 42.4% of women fulfilled the PSG diagnostic criteria for OSA. NC (38.75 cm ± 3.99 versus 40.40 cm ± 3.66, P < 0.001), WC (95.91 cm ± 12.16 versus 100.95 cm ± 11.08, P < 0.001), and BMI (25.93 ± 3.86 versus 28.07 ± 4.43, P < 0.001) were higher in patients with OSA. Most patients had mild or moderate OSA according to AHI criteria (**Table 1**).

**Figure 1.**
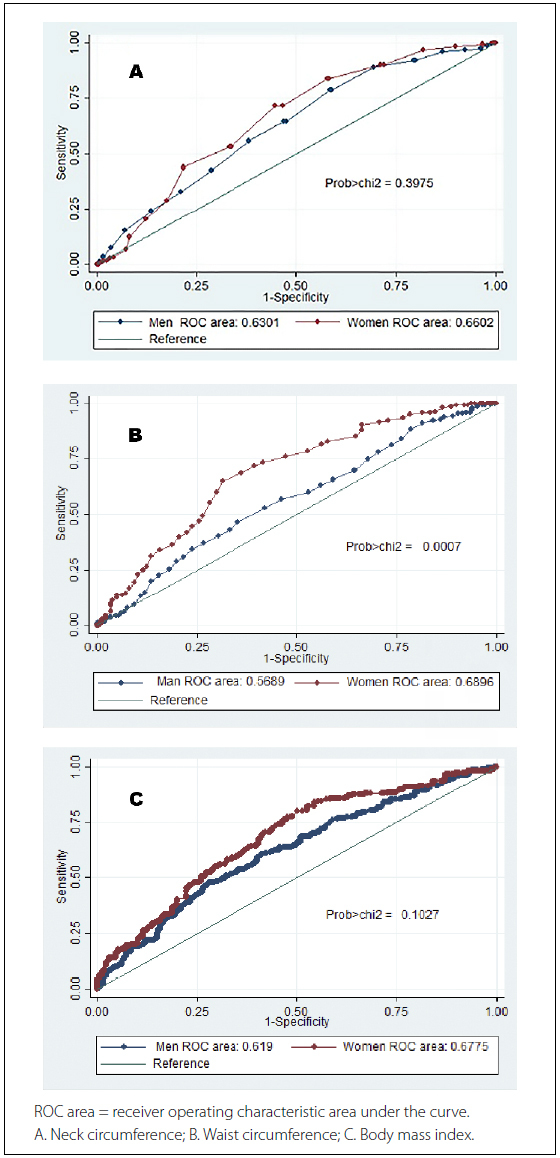
Operative characteristics curves for neck circumference, waist circumference, and body mass index, according to sex.

The discriminatory ability of NC to diagnose OSA was similar for men and women (area under the curve, AUC 0.63 versus 0.66, P = 0.39) (**Figure 1A**). However, an independent analysis of patients by age group revealed that the discriminatory ability of NC was better for women younger than 60 years than for older women (AUC 0.69 versus 0.57, P < 0.05).

The discriminatory ability of WC was better for women than for men (AUC 0.69 versus 0.57, P < 0.001) (**Figure 1B**). In the male subgroup, the discriminatory ability was better for those younger than 60 (AUC 0.62 versus 0.52, P < 0.05).

The discriminatory ability of BMI was between 0.6 and 0.7, (**Figures 1C**), with no significant differences between subgroups. All three anthropometric variables had better discriminatory ability than the positive response to the question about snoring loudly (AUC 0.57 95% confidence interval [CI] 0.54–0.60) proposed in the STOP – BANG questionnaire.

**Table 2** presents operative characteristics of NC for the optimal cut-off point and for a 40 cm cut-off point. The selected cut-off point for women was 36.5 cm (S: 56%, E: 62%) and for men, 41 cm (S: 72%, E: 55%).

**Table 2:** Operative characteristics of neck circumference for obstructive sleep apnea screening compared to polysomnography

	Operative characteristics for neck circumference ≥ 40 cm			Operative characteristics at the optimal cut-off point			

	Sensitivity (%) 95%CI	Specificity (%) 95%CI	AUC	Cut-off point (cm)	Sensitivity (%) 95%CI	Specificity (%) 95%CI	AUC
Women general	20.3 (14.6–27.1)	87.7 (82.8–91.6)	0.54 (0.50–0.57)	36.5	71.7 (64.3–78.3)	55.3 (48.7–61.8)	0.63 (0.59–0.68)
Men general	64.5 (58.1–70.5)	52.4 (46.7–58.1)	0.58 (0.54–0.62)	41.0	56.0 (49.5–62.2)	62.0 (56.1–67.2)	0.59 (0.54–0.63)
Women							
< 60	18.0 (10.9–29)	91.0 (85.1–95.1)	0.54 (0.5–0.59)	36.5	65.1 (53.5–75.3)	64.6 (56.2–72.4)	0.65 (0.58–0.71)
> 60	27.2 (18.4–37.4)	74.7 (64.5–83.3)	0.50 (0.44–0.57)	38.5	55.0 (43.5–65.4)	61.0 (49.9–71.2)	0.58 (0.50–0.65)
Men							
< 60	69.7 (61.5–77.1)	54.2 (45.7–62.5))	0.61 (0.56–0.67)	40.5	69.7 (61.5–77.1)	55.6 (47.1–63.8)	0.63 (0.57–0.68)
> 60	57.3 (47.2–67)	50.9 (43.1–58.7)	0.54 (0.48–0.60)	39.0	73.8 (64.2–82)	42.5 (34.9–50.4)	0.58 (0.52–0.63)

AUC = area under the curve; CI = confidence interval.

Selected WC cut-off points for women were 97 cm (S: 65%, E: 69%) and 99 cm (S: 53%, E: 58%) for men. **Table 3** presents the operative characteristics of WC for the optimal cut-off point and for a 90 cm cut-off point.

**Table 3.** Operative characteristics of waist circumference for obstructive sleep apnea screening compared to polysomnography

x				Operative characteristics for optimal cut-off point found in the study			

	Sensitivity (%) 95%CI	Specificity (%) 95%CI	AUC	Cut-off point cm	Sensitivity (%) 95%CI	Specificity (%) 95%CI	AUC
Women general	82.6 (76–87.9)	42.1 (35.7–48.7)	0.62 (0.58–0.66)	97.5	65.3 (57.7–72.4)	68.5 (62.2–74.4)	0.67 (0.62–0.71)
Men general	83.7 (78.4–88.1)	23.8 (19.2–28.9)	0.54 (0.50–0.57)	99.5	53.1 (46.6–59.4)	58.0 (52.2–63.4)	0.55 (0.51–0.59)
Women							
< 60	81.3 (71–89.1)	45.8 (37.5–54.3)	0.63 (0.57–0.69)	94	65.0 (53.5–75.3)	64.0 (55.5–71.7)	0.64 (0.57–0.71)
> 60	83.7 (74.5–90.6)	36.3 (26.4–47)	0.60 (0.53–0.66)	97.5	73.1 (62.9–81.8)	59.3 (48.5–69.5)	0.66 (0.59–0.73)
Men							
< 60	85.2 (78.3–90.6)	27.8 (20.6–35.8)	0.56 (0.51–0.61)	98.5	58.0 (49.4–65.9)	60.0 (51.6–67.7)	0.59 (0.53–0.64)
> 60	81.6 (72.7–88.5)	20.4 (14.5–27.3)	0.51 (0.64–0.55)	99.5	52.4 (42.4–62.4)	55.1 (47.2–62.8)	0.53 (0.47–0.59)

AUC = area under the curve; CI = confidence interval.

The BMI cut-off point with the best operative characteristics was 26.6 for men (S: 60%, E: 59%) and 26 for women (S: 71%, E: 58%) (**Table 4**).

**Table 4.** Operative characteristics of body mass index for obstructive sleep apnea screening compared to polysomnography

Operative characteristics for BMI ≥ 30				Operative characteristics for the optimal cut-off point			

	Sensitivity (%) 95%CI	Specificity (%) 95%CI	AUC	Cut-off point	Sensitivity (%) 95%CI	Specificity (%) 95%CI	AUC
Women general	31.2 (24.4–38.7)	84.3 (79–88.7)	0.58 (0.53–0.62)	26	70.5 (63.1–77.2)	56.6 (50–63)	0.64 (0.58–0.68)
Men general	23.3 (18.1–29.1)	85.2 (80.8–89)	0.54 (0.51–0.57)	26.6	60.0 (53.6–66.2)	59.0 (53.1–64.4)	0.60 (0.55–0.63)
Women							
< 60	35.0 (24.7–46.5)	81.9 (74.7–87.9)	0.58 (0.52–0.64)	25	81.3 (71–89.1)	53.5 (45–61.8)	0.67 (061–0.73)
> 60	28.0 (19.1–38.2)	87.9 (79.4–93.8)	0.57 (0.52–0.63)	27.5	57.5 (46.4–68)	70.1 (59.4–79.5)	0.64 (0.56–0.71)
Men							
< 60	23.9 (17.2–31.8)	84.0 (77–89.6)	0.49 (0.54–0.58)	26.5	60.5 (52.2–68.5)	60.0 (51.6–67.7)	0.60 (0.54–0.69)
> 60	22.3 (14.7–31.6)	86.2 (80.1–91.1)	0.54 (0.49–0.59)	26.6	61.2 (50.8–70.9)	57.2 (49.2–65)	0.60 (0.53–0.65)

BMI = body mass index; AUC = area under the curve; CI = confidence interval.

## DISCUSSION

This study is the first to determine the operative characteristics of anthropometric variables for predicting OSA in a Colombian population by comparing these characteristics to PSG as the gold standard. The results suggest that BMI, NC, and WC have moderate discriminatory ability (AUC from 0.6 to 0.7), with NC and WC having better discriminatory ability for patients under the age of 60. This study also found that the optimal cut-off points for predicting OSA were lower than those described for the North American population, but higher than those described for the Asian population.

Strikingly, OSA prevalence in this sample was relatively high (43.4%), possibly because many patients came from primary and secondary cardiovascular prevention clinics. Patients at these clinics have a higher frequency of established cardiovascular diseases and cardiovascular risk factors than other cohorts.

Previous studies have evaluated the association between anthropometric measurements and OSA in Asian and Caucasian populations. Similar to our findings, these studies reported that NC is significantly higher in patients with OSA,^10^ unlike their findings for WC and BMI, where no significant differences were found. NC has been proposed as a useful OSA screening tool and even as a tool for assessing OSA severity.^22,23^ A study in a Colombian population with 5,474 participants reported that BMI, WC, and NC were significantly higher in patients who screened positive for OSA (Berlin and STOP-Bang), but this study did not perform confirmatory tests for OSA.^15^

The present study found that NC and WC had moderate discriminatory ability for predicting OSA, with AUC values superior to those reported for the four different screening scales (Epworth, Pittsburgh, Berlin, and STOP-Bang) in the Colombian population, in which AUC values were between 0.51 and 0.56.^24^ The results of the present study suggest that easy measurements such as NC and WC may be even more useful than OSA screening questionnaires, which usually require a longer time to administer. Additionally, anthropometric measures had better discriminatory ability than the simple question about snoring. Further studies are needed to determine if the discriminatory ability of these scores (e.g., the STOP-Bang score) improves when specific cut-off points are included for each population.

A recent meta-analysis determined that obese populations in Latin America and the Caribbean had an average NC of 42.56 cm (95%CI: 41.70 cm–43.42 cm; I²: 92.40%). However, since the assessed studies used different operative definitions of NC, the meta-analysis could not determine the prevalence of patients with large NCs.^12^ This finding stresses the need to define an optimal NC cut-off point for the Colombian population.

The present study found that the optimal NC cut-off points for predicting OSA were 36.5 cm for women (S: 71.7, E: 55.3) and 41 cm for men (S: 56, E: 62), which are lower values than those reported for the North American population (41 cm for women and 43 cm for men)^8^ or those reported in Romania where the optimal cut point was 41 cm.^25^ However, the optimal cut-off points in this study were higher than those reported for the Asian population (34.5 cm for women and 38.75 for men).^9^ This suggests that the Colombian population has different ethnic characteristics, possibly associated with different obesity phenotypes.^26,27^ Cut-off points in this study are similar to those reported in Brazil (36.2 cm for women and 40.2 cm for men)^28^ and Chile (≥ 40 cm of adjusted NC, S: 77.3%, E: 67.2% for OSA diagnosis with AHI >5).^29^ The Chilean study did not differentiate between sexes. Similarly, a sub-analysis of the Sleep Heart Health Study cohort (SHHS) highlighted the NC-to-height ratio as a statistically solid alternative diagnostic method, comparable to other components of the STOP-Bang questionnaire for moderate to severe OSA screening.^30^

The findings of this study suggest similar anthropometric characteristics among Latin American populations. They also suggested that the discriminatory ability of NC may be even better in patients younger than 60 years, with AUC values of close to 0.7.

For WC, this study found that the optimal cut-off point for OSA screening was 97.5 cm and 99.5 cm for women and men, respectively, which are higher values than those established for cardiovascular risk, with acceptable discriminatory ability among women.

A strength of this study was its large sample size. This sample allowed precise optimal cut-off point calculations, even for subgroups classified by age and sex, accounting for differences in fat distribution associated with late hormonal changes in women. However, some limitations of this study should be acknowledged. This study examined a cohort with a high prevalence of cardiovascular diseases, so the possibility of generalizing the results to the broader population is limited. Further studies are required to confirm the findings of the present study. Additionally, the sample may not be fully representative of the Colombian population. However, we included patients from three different regions living at different altitudes to minimize potential bias.

## CONCLUSION

In conclusion, anthropometric measurements are simple and easy-to-use tools useful for identifying patients with OSA. The data in this study suggest that the optimal NC cut-off point in the Colombian population, and possibly in Latin America as a whole, is lower than that reported for North American populations. Complementary studies are required to determine whether including population-specific cut-off points would improve the discriminatory ability of screening scores.
